# Maternal iron intake at mid-pregnancy is associated with reduced fetal growth: results from Mothers and Children’s Environmental Health (MOCEH) study

**DOI:** 10.1186/1475-2891-12-38

**Published:** 2013-04-02

**Authors:** Ji-Yun Hwang, Ji-Yeon Lee, Ki-Nam Kim, Hyesook Kim, Eun-Hee Ha, Hyesook Park, Mina Ha, Yangho Kim, Yun-Chul Hong, Namsoo Chang

**Affiliations:** 1 Graduate School of Education, Sangmyung University, Seoul, 110-743, Korea; 2 Department of Nutritional Science and Food Management, Ewha Womans University, Seoul, 120-750, Korea; 3 Department of Preventive Medicine, College of Medicine, Ewha Womans University, Seoul, 158-710, Korea; 4 Department of Preventive Medicine, Dankook University College of Medicine, Cheonan, 330-715, Korea; 5 Department of Occupational and Environmental Medicine, Ulsan University Hospital, University of Ulsan College of Medicine, Ulsan, 682-060, Korea; 6 Department of Preventive Medicine, Seoul National University College of Medicine, Seoul, 110-799, Korea

**Keywords:** Iron, Pregnancy, Growth, Diet, MOCHE study

## Abstract

**Background:**

Iron supplementation is a common recommendation for pregnant women to prevent iron deficiency during pregnancy. There is an increasing concern about excessive iron consumption as a general iron prophylaxis by pregnant women without any due consideration about their dietary iron intake or iron status. Our present study investigated the association between total iron intake from diet and supplements and fetal growth in 337 pregnant women at mid-pregnancy in South Korea.

**Methods:**

Iron intake from diet and supplements was examined by a 24-hour recall method. Subjects were divided into three groups based on tertiles of total iron intake levels. Fetal biometry was assessed by ultrasonography at mid-pregnancy.

**Results:**

About 99% of the non-supplement users had iron intake below the recommended nutrient intake (RNI) for pregnant women (24 mg), whereas 64.9% of supplement users had iron intake above the upper level (UL) (45 mg). In the babies of mothers in the third tertile of iron intake (>17.04 mg), biparietal diameter, abdominal circumference, and femur length were lower by 0.41 cm (P =0.019), 0.41 cm (P = 0.027), and 0.07 cm (P = 0.051), respectively, than the babies of mothers in the second tertile of iron intake (11.49 ~ 17.04 mg).

**Conclusion:**

These results suggest that excessive maternal iron intake at mid-pregnancy is associated with reduced fetal growth. Iron supplementation for pregnant women should be individualized according to their iron status. Appropriate diet education is needed for pregnant women so that they can consume adequate amounts of iron from food and supplements.

## Background

Maternal iron status has been a critical factor for pregnancy outcomes, because maternal anemia as well as iron deficiency increases the risk of adverse pregnancy outcomes such as preterm delivery [1] and low birth weight [2]. Although iron supplementation is a common recommendation for pregnant women to prevent iron deficiency during pregnancy, the beneficial effects of general iron supplementation on pregnancy outcomes are a controversial issue [3,4].

Until the past 10 years or so, the risk of iron deficiency in Korean pregnant women was high with a prevalence of ~20% [5,6] and a number of reports are available on inverse association between maternal iron status and pregnancy outcomes [7-10]. Recently, there has been an increasing concern that pregnant women in Korea might be consuming excessive iron from supplements without considering their dietary iron intake or iron status. A recent survey has reported that 30 – 40% of Korean women in their childbearing age consumed one or more dietary supplements [11] and 47.3% of supplement users took supplements on their own, without any prescription [12]. The amount of iron intake from supplements alone was already twice the level of the estimated average requirement (EAR, 18.5 mg/d) for pregnant women in Korea [13].

The relationship between maternal hemoglobin (Hb) concentrations and birth weight has been reported to be of U-shaped [14,15]. Recent studies have reported negative associations between high Hb concentrations (> 130 g/L) and pregnancy outcomes in diverse populations in China [16,17], US [14,18], Sweden [19], and Korea [4,20]. A possible explanation for this negative association is that excessive iron could lead to oxidative damage [21] and decrease in the absorption of copper and zinc [22], which are the important micronutrients for fetal growth.

Previous studies have reported that general iron supplementation have harmful effects on pregnancy outcomes [23,24]. However, those studies have not taken into account the levels of iron intake from food. Even though previous studies in the U.S., [25,26] and England [27,28] have reported no relation between iron intake from both food and supplements and pregnancy outcomes, fetal growth during pregnancy has not been considered. Therefore, the present study was performed to investigate the association between maternal iron intake from both food and supplements at mid-pregnancy. Fetal biometry was assessed by ultrasonography at mid-pregnancy. To the best of our knowledge, none of the studies have employed ultrasonography, which is a reliable measurement for fetal growth, to assess the relation between maternal iron intake and fetal growth during pregnancy.

## Methods

### Study population

The present investigation was undertaken as a part of a prospective cohort study of the Mothers and Children’s Environmental Health (MOCEH) in South Korea, established in 2006. Details of the methods of the MOCEH study have been described previously [29]. The three local centers involved in the study are located in Seoul (a metropolitan area), Ulsan (an industrial area), and Cheonan (a medium-sized urban area). We recruited pregnant women who were at 12–28 weeks of gestation and registered at a local center for participation in the MOCEH study from August 2006 to October 2009. The study sample comprised of pregnant women at mid-pregnancy of a normal pregnancy, who voluntarily consented to participate in this study after the purpose of the investigation, was explained to them. A total number of 1563 (537 from Seoul, 491 from Cheonan, and 535 from Ulsan) pregnant women were recruited after excluding the cases with twins (*n* = 25) or spontaneous abortion (*n* = 22). Of these, we excluded those who have not yet delivered babies (n = 399), whose pregnancy lasted less than 37 or more than 42 weeks (*n* = 63), or who had complications associated with pregnancy (diabetes or hypertension) (*n* = 29). Among the 1072 subjects, 85 subjects who were without any dietary intake data and 3 subjects who had energy consumption of < 500 kcal/d or > 4000 kcal/d were excluded from the study. Of the 984 subjects, 296 in whom the data related to fetal biometric measurements (biparietal diameter, abdominal circumference and femur length) was absent at mid-pregnancy were excluded. In order to investigate the later effect of maternal nutrients on fetal growth, 353 subjects whose diet intake data at mid-pregnancy were assessed after the ultrasonography was performed at mid-pregnancy were also excluded. Thus, a total of 337 women (51 from Seoul, 123 from Cheonan, and 163 from Ulsan) were included in this study. The general characteristics did not differ significantly between included and excluded subjects (data now shown). The study protocols and consent forms were approved by three institutional review boards: Ewha Woman’s University School of Medicine, Dankook University Hospital, and Ulsan University Hospital.

### Measurements

The participants were interviewed by a trained interviewer during their visits to local university hospitals, local clinics, and community public health centers. Information on demographic factor and socioeconomic factors including age, height, weight, education, family income, exercise, smoking behavior, alcohol drinking were obtained by individual interviews. Pre-pregnancy body mass index (BMI) was calculated as weight (kg) divided by height (m^2^) by employing self-reported height and weight values before conception. The sociodemographic factors were categorized as follows: education level (< high school, < university and ≥ university), family monthly incomes (< US$ 2000, US$ 2000–4000, and > US$ 4000), passive exposure to cigarette smoke (yes/no) and parity (n).

As indices for fetal biometry, the biparietal diameter (BPD), abdominal circumference (AC), and femur length (FL) were assessed by ultrasonography at mid-pregnancy. Fetal biometry measurements were carried out at Ewha Womans University Hospital by a certified ultrasonographer. The gestational age determined by ultrasound measurement was obtained from medical records.

Dietary intake at mid-pregnancy was estimated at individual medical centers by trained interviewers by employing a 24-hour recall method. The subjects were asked to describe all the foods and beverages, which they had consumed in the past 24 hours before the interview. Extra questions were put forth for obtaining details about some items such as snacks, drinks, or extra salt in the case of subjects, who hardly remembered about what they had eaten in the past 24 hours. The indicated iron intakes incorporated both food and supplements sources. Iron intake from a 24-hour recall was assessed by using a computerized nutrient intake analysis program (CAN-Pro 3.0, Korean Nutrition Society, Seoul, Korea). The consumption of nutritional supplements was investigated on the basis of self-reporting on the same day, when the dietary questionnaire was administered. Information on type and brand name of supplements and amounts and frequency of its use was also directly gathered from the participants. Maternal total iron intake incorporated both dietary iron and in the form of supplements.

### Statistical analysis

The data are expressed as mean ± SD values (continuous variables) or as numbers and percentages (categorical variables). Maternal iron intake and urinary cotinine levels were log-transformed due to skewed distribution. In order to examine the difference in fetal biometry based on their iron intake level, the subjects were divided into tertiles of total iron intake levels (<11.49 mg, 11.49 ~ 17.04 mg, >17.04 mg). The differences in iron intake between supplement users and non-supplement users were analyzed by using student’s *t*-test. The difference in fetal biometry according to maternal total iron intake levels was tested by using the generalized linear model (GLM) test after controlling for confounding factors [gestational age at time of ultrasound measurement, maternal age, maternal height, urinary cotinine level, pre-pregnancy BMI, parity, family monthly income, sex of babies and energy intake]. All the statistical tests were two-tailed and a P value of < 0.05 was considered to be significant. Statistic analyses were performed with the SPSS statistical package (version 12.0, SPSS, Chicago, IL, USA) and SAS software (SAS 9.1, SAS Institute, Cary, NC, USA).

## Results

### Study population characteristics

The participants had a mean age of 29.9 ± 3.6 years and pre-pregnancy BMI of 21.9 ± 3.5 kg/m^2^ (Table 1). Approximately, 28.8% of subjects took iron containing dietary supplements. The mean gestational age at the time of ultrasound measurement, fetal biparietal diameters, abdominal circumferences, and femur lengths were 20.0 ± 3.4 weeks, 4.8 ± 1.1 cm, 16.6 ± 3.8 cm and 3.3 ± 1.0 cm, respectively.

**Table 1:** **General characteristics of pregnant women and fetuses**^**1**^

**Variables**	**n**	**Values**
*Pregnant women*		
Age, *y*	327	29.9 ± 3.6
Height, *cm*	324	160.8 ± 4.8
Pre-pregnancy weight, *kg*	325	56.5 ± 9.7
Pre-pregnancy BMI^2^, *kg/m*^*2*^	323	21.9 ± 3.5
Education, *n* (%)	337	
< High school		99 (29.4)
< University		59 (17.5)
≥ University		163 (48.4)
No-response		16 (4.7)
Family income, US$/mo, *n* (%)	337	
< 2000		95 (28.2)
2000 - 4000		174 (51.6)
> 4000		43 (12.8)
No-response		25 (7.4)
Passive smoker, *n* (%)	337	
No		41 (12.2)
Yes		268 (79.5)
No-response		28 (8.3)
Urinary cotinine level, *μg/g creatinine*	316	71.8 ± 444.2
Supplement users, *n* (%)	337	
No		240 (71.2)
Yes		97 (28.8)
Parity	337	
0		180 (53.4)
≥1		147 (43.6)
No-response		10 (3.0)
Fetuses		
Sex	323	
Girls, *n* (%)		164 (48.7)
Boys, *n* (%)		159 (47.2)
Gestational age, *wk*	337	20.0 ± 3.4
Biparietal diameter, *cm*	334	4.8 ± 1.1
Abdominal circumference, *cm*	231	16.6 ± 3.8
Femur length, *cm*	297	3.3 ± 1.0

^1^ Values are mean ± SD or *n* (%).

### Iron intake of subjects

The average iron intake of subjects was 30.9 ± 39.2 mg (12.5 ± 3.9 mg from foods and 18.4 ± 38.8 mg from supplements) (Table 2). Although the total iron intake of supplement users was six times greater than that of non-supplement users (76.9 ± 48.5 mg vs. 12.4 ± 3.8 mg), there was no marked difference in iron intake from food sources between the two groups. About 99% of the non-supplement users had iron intake below the RNI (24 mg), whereas 64.9% of supplement users had iron intake above the UL (45 mg).

**Table 2:** **Iron intake at mid-pregnancy according to intake of supplements and total iron intake levels at mid-pregnancy**^**1**^

	**All subjects (*n =*** **337)**		**Supplement users (*n =*** **97)**		**Non-supplement users (*n =*** **240)**	
	**n (%)**	**Mean ± SD**	***n *(%)**	**Mean ± SD**	***n *(%)**	**Mean ± SD**
Food,^3^ mg		12.5 ± 3.9		13.0 ± 3.9		12.4 ± 3.8
Supplements,^4^ mg		18.4 ± 38.8		63.8 ± 48.3		-
Total iron intake^5^, *mg*		30.9 ± 39.2		76.9 ± 48.5^a2^		12.4 ± 3.8^b^
< 24	256 (76.0)	12.6 ± 3.9	18 (18.6)	17.9 ± 4.1^2a^	238 (99.2)	12.2 ± 3.6^b^
24 ~ < 45	18 (5.3)	33.9 ± 6.2	16 (16.5)	34.8 ± 5.9^a^	2 (0.8)	26.8 ± 3.5^a^
≥ 45	63 (18.7)	104.4 ± 37.4	63 (64.9)	104.4 ± 37.4	0	
1^st^ tertile (< 11.49)	112 (33.3)	9.3 ± 1.7	1 (1.0)	-	111 (46.3)	9.2 ± 1.7
2^nd^ tertile (11.49 ~ 17.04)	113 (33.5)	14.0 ± 1.6	8 (8.3)	15.1 ± 1.7^a^	105 (43.8)	13.9 ± 1.5^b^
3^rd^ tertile (>17.04)	112 (33.2)	69.7 ± 48.7	88 (90.7)	83.2 ± 46.4^a^	24 (10.0)	20.2 ± 3.0^b^

^1^ Values are mean ± SD or *n* (%).

^2^ Significantly different by student’s *t*-test; Values with different superscript letters within a row are significantly different between supplement uses and non-supplement users (*P* < 0.05).

^3^ Iron intake from food sources only.

^4^ Iron intake from supplement sources only.

^5^ Total iron intake from both food and supplement sources.

### Fetal biometry and fetal growth according to iron intake from different sources

Fetal biometry and growth were not different according to the tertile of dietary iron intake adjusting for confounding factors including iron intake from supplements (data now shown). However, in the babies of mothers with iron supplementation, biparietal diameter and abdominal circumference were greater by 0.09 cm (P =0.012) and 0.39 cm (P = 0.017), respectively, than the babies of mothers without iron supplementation after adjusting for confounding factors including dietary iron intake (Table 3).

**Table 3:** Fetal biometry at mid-pregnancy according to supplemental iron intake level at mid-pregnancy

		**Iron supplement use**				
	**Total**	**Supplement users (> 0)**		**Non-supplement users (= 0)**		
**Fetal growth**^**1**^	**n**	**β (SE)**	***P*-value**	**β (SE)**	***P*-value**	**R**^**2**^
Biparietal diameter, *cm*	294	1		0.092 (0.036)	0.012	0.941
Abdominal circumference, *cm*	212	1		0.391 (0.163)	0.017	0.923
Femur length, *cm*	265	1		0.057 (0.032)	0.077	0.946

^1^ Adjusted for gestational age at the time of ultrasound measurement, maternal age, maternal height, urinary cotinine level (ln), pre-pregnancy BMI, parity, family monthly income, sex of babies, energy intake (ln) and dietary iron intake (ln).

### Fetal biometry and fetal growth according to tertiles of iron intake levels

In the babies of mothers in the third tertile of iron intake (>17.04 mg), biparietal diameter, abdominal circumference, and femur length were lower by 0.41 cm (P =0.019), 0.41 cm (P = 0.027), and 0.07 cm (P = 0.051), respectively, than the babies of mothers in the second tertile of iron intake (11.49 ~ 17.04 mg) (after adjusting for confounding factors) (Table 4).

**Table 4:** Fetal biometry at mid-pregnancy according to total iron intake level at mid-pregnancy

		**Total iron intake (mg)**						
	**Total**	**1**^**st **^**tertile (< 11.49)**		**2**^**nd **^**tertile (11.49 ~ < 17.04)**		**3**^**rd **^**tertile (≥ 17.04)**		
**Fetal growth**^**1**^	**n**	**β (SE)**	***P*-value**	**β (SE)**	***P*-value**	**β (SE)**	***P*-value**	**R**^**2**^
Biparietal diameter, *cm*	294	−0.03 (0.04)	0.488	1		−0.09 (0.04)	0.019	0.941
Abdominal circumference, *cm*	212	−0.01 (0.22)	0.958	1		−0.41 (0.18)	0.027	0.922
Femur length, *cm*	265	−0.01 (0.04)	0.877	1		−0.07 (0.04)	0.051	0.946

^1^ Adjusted for gestational age at the time of ultrasound measurement, maternal age, maternal height, urinary cotinine level (ln), pre-pregnancy BMI, parity, family monthly income, sex of babies and energy intake (ln).

## Discussion

We found that the fetuses of pregnant women with iron intake in the 3^rd^ tertile (>17.04 mg) had a significantly lower a biparietal diameter, abdominal circumference, and femur length than the fetuses of mothers in the second tertile of iron intake (11.49 ~ 17.04 mg). About 90% of the highest tertile were iron supplemental users and all the mothers who consumed iron above the UL (45 mg) took supplements containing iron. The average iron intake of supplement users was 76.9 ± 48.5 mg, which was three times higher than the RNI (24 mg) and the source was mainly from the supplements; while the iron intake from food sources was only 13.0 ± 3.9 mg which was lower than the EAR (18.5 mg). Fetal biometry and growth were not different according to the dietary iron intake adjusting for iron supplementation use, but were greater in the babies of mothers with iron supplementation than the babies of mothers without iron supplementation adjusting for dietary iron intake. These results indicate that supplemental iron in pregnancy is needed for fetal growth but also indicate that excessive consumption of iron, mainly from the supplements at mid-pregnancy may have adverse effects on fetal growth at mid-pregnancy.

Several studies have reported adverse effects of iron supplements on pregnancy outcomes. Ziaei et al. [23] reported that SGA birth rate and the number of pregnant women with hypertension were significantly higher in the iron-supplemented group (50 mg/d) when compared with the non-iron-supplemented group (with Hb level of 132 g/L and above). In their study, no other differences were observed in the pregnancy outcomes between both the groups. A randomized trial has revealed that a higher frequency of infant hospitalization due to convulsions was observed in the routine iron supplemented group (100 mg/d) when compared with the selective iron supplemented group [24]. The selective iron supplemented group was given iron supplementation if the hematocrit was < 30% and the mother was diagnosed to be anemic. These results indicate that a high dose of general iron prophylaxis without due consideration about the maternal iron status might have a negative influence on fetus and infant health.

The mechanisms that explain these adverse effects due to excessive iron intake have not yet been clearly established; however, several potential mechanisms have been suggested. Primarily, excessive iron intake could cause free-radical damage during pregnancy [30-32]. Pregnancy itself is vulnerable to oxidative stress [31]. Free iron catalyzes the transformation of hydrogen peroxide to a hydroxyl radical via the Fenton Reaction. Studies have shown that the levels of free radicals were higher in pregnant women who consumed iron supplements in Estonia (36 mg/d) [33] and in the US (19 mg/d) [34] than those without any consumption of supplements. The free radicals have been reported to damage cellular DNA, proteins and lipids [35]. According to Takagi et al., the level of oxidative stress and redox-related molecules was higher in the placenta in preeclampsia and IUGR than in normal pregnancy [36]. Secondly, previous studies have reported that iron supplements could interrupt the absorption of zinc and copper in pregnant women with Hb ≥132 g/L [37,38] or with normal Hb levels [39,40]; the reason being competitive absorption of iron, zinc, and copper from the small intestine via divalent metal transporter. Moreover, a high dose of iron supplements (>30 mg/day) can frequently lead to gastrointestinal problems such as constipation, diarrhea, nausea and vomiting [41].

Our results indicate that iron intake from food in pregnant women was inadequate but supplement users consumed an excessively high amount of iron from the supplements. It is evident from the previous reports that iron requirements during pregnancy cannot be satisfied by iron from foods alone and iron supplementation is necessary during pregnancy [42] and is helpful for adequate fetal growth. In our study, total iron intake was lower than the recommended level in women who did not consume iron supplements and amongst the women with iron intake below the RNI (24 mg), only 7% of them took iron supplements. In contrast, supplement users had iron intake of 76.9 ± 48.5 mg, which was three times higher than the RNI (24 mg) and the iron intake exceeded the UL in 65% of the supplement users. Some supplement users also took prescribed iron supplements in addition to over-the-counter multi-vitamin supplements, while others took higher dose of iron supplements than the prescribed dosage. This raises a potential problem that some pregnant women, non-anemic, non-iron deficient, might consume unnecessarily high amounts of general iron prophylaxis during pregnancy.

In the case of non-anemic pregnant women in Mexico, high dose of iron-supplementation (60 mg/day) was related to high rates of hemoconcentration (Hb > 145 g/L), which was further associated with a relatively high risk of low birth weight and premature delivery [43]. However, a low dose of iron supplement (30 mg/d) was significantly associated with a higher mean birth weight, a lower incidence of low-birth-weight infants and lower incidence of preterm low-birth-weight infants in 513 iron-replete non-anemic pregnant women in the U.S. Daily supplementation [44]. Therefore, a low dose of iron supplements (30 mg/d or 120 mg/wk) may be more beneficial for fetal growth than a high dose of iron and proper advice about iron supplementation should be provided by a nutritionist or health professionals to prevent excessive iron intake by pregnant women.

Iron is necessary for normal fetal growth and development and the physiologic iron requirements during the second half of pregnancy cannot be achieved by dietary iron only. Iron supplementation during pregnancy consistently increase serum ferritin and hemoglobin and decrease the prevalence of iron deficiency anemia. With consideration of negative influences of iron such as increased oxidative stress and competitive absorption with other divalent metals during pregnancy, the lowest effective dose of iron supplementation based on women’s iron status should be recommended. Milman [42] suggested that individual iron prophylaxis according to serum ferritin status, indicative of risk for iron deficiency, should be preferred to general prophylaxis. In addition, bioavailability of iron from animal foods is higher than iron from plant foods due to high composition of hem iron and meat factors. Components in food such as phytate and polyphenols reduce the absorption of both dietary and supplementary iron. Maximum absorption of ferrous iron from supplementation can obtained when the tablets is taken between the meals rather than at or after a meal [45].

Our present study was associated with several limitations. Firstly, maternal iron status in blood was not assessed in the present study although maternal hematological parameters such as, Hb and hematocrit levels are important ones to be considered when we study the effect of maternal iron intake on fetal growth [46]. Secondly, the employed dietary intake and supplements were investigated for only a single time. A 24-hour recall may be insufficient to postulate that the values are from a usual daily intake due to large intra-individual variability. Although measurements of dietary intake based on a single 24–hour recall per individual of the large sample size can provide a good probable estimate of the mean for a study population, the standard deviation will be greatly overestimated. Furthermore, the random within-person variation may reduce the strength of associations under the study, possibly to the point of being undetectable. However, trained dietitians employed standard protocols in helping the subjects in reporting their daily diet to minimize plausible errors. Third, recruitment from voluntary participation as well as non-participation or non-responses might have introduced a selection bias. But, general characteristics did not differ significantly between included and excluded subjects and high recruitment rate in main centers of the study minimizes the chance that results were distorted by selection bias. To the best of our knowledge, this is the first study to investigate the association between maternal total iron intake and fetal growth in a relatively large sample, as assessed by ultrasonography. In addition, this work had certain advantages over previous studies because it was a multi-center prospective cohort study that utilized a strict protocol. Moreover, possible confounders such as gestational age at the time of ultrasound measurement, maternal age, maternal height, urinary cotinine level, pre-pregnancy BMI, parity, family monthly income, sex of babies and energy intake were all accounted for the analyses.

## Conclusion

In conclusion, excessive maternal iron intake at mid-pregnancy is associated with reduced fetal growth. Our results suggest that iron supplements of a low dose instead of high dose may be a better option for the pregnant women. Further studies are needed to assess the risks or benefits of total iron intake according to maternal iron status in pregnant women, and to investigate the relationship of maternal iron intake with birth outcomes and postnatal growth in the present study group.

## Competing interests

All authors, JY Hwang, JY Lee, KN Kim, H Kim, EH Ha, H Park, M Ha, H Kim, YC Hong, and N Chang, declare that they have no competing interests.

## Authors’ contributions

NC designed the research; JYL, KNK, HK, EHH, HP, MH, YK, and YCH conducted the research; JYL analyzed the data; JYH, JYL, and NC wrote the manuscript; and NC was primarily responsible for the final content. All the authors read and approved the final manuscript.
